# Comprehensive Identification of *CPP* Gene Family Members in *Panax ginseng* and Expression Analysis of *PgCPP* and Key Protopanaxadiol Ginsenoside Biosynthesis Genes in Response to MeJA

**DOI:** 10.3390/biology15131063

**Published:** 2026-07-03

**Authors:** Bohan Yan, Hexuan Li, Dazhun Guan, Yu Zhang, Kexin Zhang, Shuang Li, Kangyu Wang

**Affiliations:** 1 College of Life Science, Jilin Agricultural University, Changchun 130118, China; m18750271001@163.com (B.Y.); lihexuan@mails.jlau.edu.cn (H.L.); guandazhun123456@163.com (D.G.); yuzi_zhang0913@163.com (Y.Z.); zkx12302023@163.com (K.Z.); 2 Jilin Engineering Research Center Ginseng Genetic Resources Development and Utilization, Jilin Agricultural University, Changchun 130118, China; 3 Teaching and Scientific Research Base Management Office, Jilin Agricultural University, Changchun 130118, China

**Keywords:** *CPP* gene family, *Panax ginseng*, protopanaxadiol-type ginsenosides, systematic evolution, cis-acting element

## Abstract

The Cysteine-rich Polycomb-like Protein (*CPP*) gene family is a class of transcription factors containing conserved CXC domains that are widely involved in the regulation of plant growth and development, cell division, and stress responses. Based on the ginseng genome and transcriptome database, all members of the *PgCPP* gene family in *Panax ginseng* were systematically identified, and comprehensive bioinformatics analyses, including phylogenetic, conserved domain, chromosomal localization and collinearity, cis-acting element, and expression pattern analyses, were conducted. In this study, we identified 44 *PgCPP* gene family members in ginseng, which were unevenly localized on multiple chromosomes. The phylogenetic tree divided them into three subfamilies, with members in the same subfamily being highly conserved. Conserved domain analysis revealed that all PgCPPs contain typical CXC motifs. Cis-acting elements were abundant in light response, hormone responses (abscisic acid, methyl jasmonate, salicylic acid), and stress response elements. Expression heatmaps demonstrated that different members have specific expression patterns across different ages, tissues, and species. After treatment with MeJA, transcriptional suppression of *PgCPP03-4* and *PgCPP03-13* was observed, and their expression levels demonstrated significant negative correlations with the contents of six protopanaxadiol-type ginsenosides. These findings suggest that *PgCPP03-4* and *PgCPP03-13* may act as negative regulators of protopanaxadiol-type ginsenoside biosynthesis within the MeJA signaling pathway. This systematic characterization and identification of the *CPP* gene family members in *P. ginseng* establishes a foundational framework for future functional validation and molecular breeding initiatives.

## Simple Summary

The *CPP* gene family members in ginseng were identified and systematically analyzed, allowing for the identification of *PgCPP* genes potentially involved in the regulation of protopanaxadiol-type ginsenoside biosynthesis. Following treatment with MeJA, the expression of the two candidate *PgCPP* genes (*PgPP03-4* and *PgPP03-13*) decreased significantly, thereby possibly inhibiting protopanaxadiol-type ginsenoside biosynthesis.

## 1. Introduction

The Cys-rich Polycomb-like Protein (*CPP*) gene family is a class of plant-specific transcription factors, with its core feature being the highly conserved CXC (Cys-X-Cys) domain. The *CPP* gene family was first discovered in corn [1] and was named for its high cysteine content and certain similarities to the polycomb protein in animals [2,3]. Unlike animal polycomb proteins—which are involved in epigenetic regulation—*CPP* gene family members in plants are usually located in the nucleus and serve as the main transcriptional regulators involved in processes such as cell growth and development, cell division, and abiotic stress responses [4]. For example, in a study investigating the mechanisms by which *CPP* genes regulate rice leaf development, the OsCPP1 protein was found to be localized in the nucleus and to influence the formation and expansion of leaf primordia by regulating the cell division cycle and the transcription of related downstream target genes, demonstrating its central role in leaf morphogenesis [5]. In research on the wheat *CPP* gene family, *TaCPP1* and *TaCPP3* have been shown to rapidly activate the expression of downstream antioxidant enzyme genes (e.g., superoxide dismutase and catalase) through induction, eliminating excess reactive oxygen species and mitigating lipid peroxidation damage. Furthermore, *TaCPP1* overexpression significantly enhanced the photosynthetic efficiency and survival rate of transgenic wheat under drought and high-temperature stress conditions, thereby improving its overall tolerance to multiple stressors [6]. Although the functions of the *CPP* gene family have gradually been elucidated in some model plants, their potential roles in ginseng have not yet been investigated.

*Panax ginseng* is a perennial herbal medicinal plant belonging to the Araliaceae family [7] known as the “King of Herbs,” with a long medicinal history in China and East Asia [8]. The main pharmacologically active components of ginseng are ginsenosides, which belong to the triterpene saponin class and show multiple pharmacological activities, including anti-inflammatory, antioxidant, antitumor, immunomodulatory, and neuroprotective effects [9]. The biosynthetic pathways of ginsenosides have been systematically studied—mainly those involving mevalonic acid (MVA) and 2-C-methyl-D-erythritol-4-phosphate (MEP)—as well as their modification by cyclization, hydroxylation, and glycosylation of squalene oxide to form various saponin monomers [10]. Among these, protopanaxadiol (PPD) and protopanaxatriol (PPT) are the main framework monomers [11]. Although key enzyme genes regulating these synthetic pathways, such as *MYB* [12] and *WRKY* [13], have been identified, no studies to date have assessed whether the *CPP* family genes in ginseng are involved in the transcriptional regulation of ginsenoside biosynthesis.

The continuous improvement of ginseng genomic and transcriptome data provides an important foundation for the genome-wide identification of *CPP* gene family members and relevant functional studies. Based on the ginseng genome and transcriptome database, we systematically identified 44 *PgCPP* gene family members with the conserved CXC domain in *P. ginseng*. Comparing the reported chromosomal localization of *CPP* gene family genes across different plant species, 11 members of the *OsCPP* family in rice are localized to only 9 out of 12 chromosomes, with 3 chromosomes remaining unlocalized [5]; meanwhile, in maize, 12 members of the *ZmCPP* family are distributed across 6 chromosomes—predominantly on Chr1 and Chr3—while only 1–2 genes are localized on each of the other chromosomes, exhibiting a highly uneven distribution [1]. These findings demonstrate that *CPP* genes tend to exhibit an irregular polychromosomal distribution pattern. Using bioinformatics methods, the sequence characteristics, phylogenetic relationships, chromosomal localization, collinearity, conserved motifs, promoter cis-acting elements, and expression patterns in different plant tissues, ages, and cultivars were analyzed. Further co-expression network analysis identified candidate *PgCPP* genes related to key enzyme genes involved in ginsenoside synthesis. Recent studies have further elucidated the integrated mechanisms of plant hormone signaling networks in regulating secondary metabolite accumulation; for instance, auxin signals interact with various stress signaling pathways in plants to modulate the integration of environmental signals and the accumulation of secondary metabolites [14]. MeJA treatment can induce the differential expression of relevant genes in ginseng, thereby regulating the anabolic metabolism of ginsenosides and altering their accumulation levels [15]. Therefore, taking MeJA treatment of ginseng adventitious roots as the experimental system, the expression dynamics of the genes *PgCPP03-4* and *PgCPP03-13* and their correlations with the contents of six protopanaxadiol-type ginsenosides (Rb1, Rc, Rb2, Rd, Rg2, and Rb3) were analyzed, providing a theoretical basis and candidate gene resources for the molecular breeding and genetic-engineering-based improvement of high-saponin-yield ginseng cultivars.

## 2. Materials and Methods

### 2.1. Comprehensive Identification of the CPP Gene Family Members in Ginseng

The *CPP* gene family members were systematically identified utilizing ginseng genome and transcriptome datasets [16,17]. Initial domain screening was performed using the Hidden Markov Model (HMM) profile of the CPPs (Pfam number: PF03638) obtained from the InterProScan (https://www.ebi.ac.uk/interpro/, accessed on 25 March 2026) database. We screened against the profile using HMMER version 3.2 (http://hmmer.org/download.html, accessed on 25 March 2026) with an E-value of 1.0 × 10^−5^. Subsequently, putative protein candidates underwent conserved domain validation employing NCBI’s Conserved Domain Database (CDD) version 3.20 search tool (https://www.ncbi.nlm.nih.gov/Structure/cdd/wrpsb.cgi, accessed on 2 April 2026) with default parameters. The final *PgCPP* gene family members were designated identifiers (*PgCPP01*–*06* with corresponding subtypes) based on CXC domains in the relevant proteins.

### 2.2. Phylogenetic Analysis and Gene Structure Analysis of the PgCPP Gene Family

To classify the identified *CPP* gene family members, those in maize (*Zea mays*), *Arabidopsis* (*Arabidopsis thaliana*), tomato (*Solanum lycopersicum*), soybean (*Glycine max*), and wheat (*Triticum aestivum*) were taken as a reference. We performed a phylogenetic analysis on 74 protein-coding genes from these five species and ginseng. The phylogenetic tree was generated using the maximum likelihood (ML) method in the MEGA version 11.0 software. The tree diagram was then improved for viewing purposes using the online tool iTOL (https://itol.embl.de/itol_account.cgi, accessed on 6 April 2026). We also used the MEME version 5.5.9 software to study conserved motifs, with motif lengths set between 10 and 50 amino acids.

### 2.3. Chromosomal Localization and Collinearity Analysis of the PgCPP Gene Family Members

We investigated the localization of the *PgCPP* gene family members within the ginseng genome [17]. The BLASTN version 2.14.0 tool facilitated comparison of the *PgCPP* gene family sequences with the ginseng genome, requiring a sequence consistency of at least 95%, an alignment length of no less than 200 bp, and an E-value not exceeding 1.0 × 10^−100^. Subsequently, the MG2C online tool (http://mg2c.iask.in/mg2c_v2.1/index.html, accessed on 17 April 2026) was utilized to visualize the chromosomal positioning of the *PgCPP* genes. Finally, TBtools II version 2.0 was employed to illustrate the chromosomal distribution of *PgCPP* genes and perform a collinearity analysis [18].

### 2.4. PgCPP Gene Expression Pattern Analysis in Ginseng

This study analyzed the expression patterns of *PgCPP* genes [16]. The dataset comprises TPM expression data for *PgCPP* gene family members at four different ages (5, 12, 18, and 25 years), 14 different tissues of four-year-old ginseng plants (leaf peduncle, leaflet pedicel, fruit peduncle, fruit flesh, main root epidermis, seed, fruit pedicel, leg roots, main root cortex, rhizome, arm roots, fiber roots, leaf blade, and stem), and roots from 42 landraces ginseng cultivars (S1–S42). Concurrently, TBtools II version 2.0 was employed to generate heatmaps, thereby providing a visual representation of *PgCPP* gene member expression patterns [18].

### 2.5. PgCPP Gene Co-Expression Network Analysis in Ginseng

To study the expression of *PgCPP* genes in 42 landrace ginseng cultivars, the Pearson correlation coefficients for *PgCPP* gene expression were calculated using SPSS version 23.0. Then, the BioLayout Express 3D version 3.4 software was used to construct a *PgCPP* gene co-expression network (*p* < 0.05). The gene expression correlations were then visualized using the online platform CNSKnowall (https://cnsknowall.com/#/Home/HighAll/, accessed on 20 April 2026). This analysis clarified the expression patterns of *PgCPP* genes in different cultivars and their possible functional links.

### 2.6. Cis-Regulated Element Analysis of the PgCPP Gene Family Promoters

To investigate the potential biological functions of the *PgCPP* gene family members, promoter sequences located upstream of the initial codon were selected based on the chromosomal localization of the *PgCPP* genes. These sequences were subjected to analysis using the online tool PlantCARE (https://bioinformatics.psb.ugent.be/webtools/plantcare/html/, accessed on 25 April 2026), in order to identify cis-acting elements [19]. The analysis identified cis-acting elements associated with hormone response functions. Subsequently, the Plant-CARE Result Classify module of TBtools II version 2.0 was employed to visualize these components, elucidating the potential regulatory mechanisms of the *PgCPP* gene family within hormone signaling networks [18].

### 2.7. Screening of PgCPP Candidate Genes Involved in Ginsenoside Biosynthesis

Gene expression patterns were analyzed using expression data for *PgCPP* genes and 20 key enzyme genes associated with ginsenoside biosynthesis, including *CAS_17*, *SS_1*, *DS_3*, *SE2_1*, *UGT71A27_2*, *SE2_2*, *AS_14*, *CYP716A53v2_1*, *DS_1*, *FPS_22*, *SE2_4*, *AS_6*, and *CAS_14* genes [20,21]. A Pearson correlation analysis was performed using SPSS version 23.0 to investigate the relationships between the expression levels of the *PgCPP* genes and the selected key enzyme genes for ginsenoside biosynthesis. The analysis results were visualized using the CNSKnowall tool (https://cnsknowall.com/#/Home/HighAll/, accessed on 28 April 2026) and the online tool Prism (https://www.graphpad.com/, accessed on 28 April 2026).

### 2.8. PgCPP Candidate Gene Expression Analysis After MeJA Treatment

In this study, 1.0 g of ginseng adventitious roots were inoculated into a 250 mL conical flask containing 150 mL Murashige and Skoog (MS) liquid medium (Solarbio, Beijing, China) and cultured in a shaker at 22 °C and 110 rpm for 21 days. Then, 200 µM MeJA (Solarbio, Beijing, China) (the optimal concentration verified at our laboratory) was used to treat the ginseng adventitious roots with treatment times of 0, 6, 12, 24, 36, 48, 60, 72, 84, 96, 108, and 120 h, with three biological replicates at each treatment time, and the untreated group at 0 h serving as the control group (CK). After MeJA treatment, 0.1 g of ginseng adventitious root material was collected, and total RNA was extracted using the Trizol method. In this study, the *Actin 1* gene was used as the reference gene [22], and all the primers used and their sequences are listed in Table 1. The fluorescence quantitative PCR (qRT-PCR) experiment was performed using the 7500 real-time fluorescence quantitative PCR system (ABI, Foster City, CA, USA) with the UltraSYBR one-step qRT-PCR kit (Low ROX) (CWBIO, Beijing, China), following the manufacturer’s instructions. To ensure the accuracy of the results, each experiment was set with three biological and three technical replicates, and the obtained data were analyzed using the 2^−ΔΔCt^ method.

### 2.9. Correlation Analysis of PgCPP Gene Expression and Protopanaxadiol-Type Ginsenoside Content

In this study, we analyzed 42 ginseng samples using high-performance liquid chromatography (HPLC) to determine the contents of six protopanaxadiol-type ginsenosides (Rb1, Rc, Rb2, Rd, Rg2, and Rb3). A one-way ANOVA was performed on gene expression level data at different time points, using the untreated samples as a control group. The ginseng rhizomes remaining after sampling were cleaned of impurities, dried at low temperature until reaching a constant weight, and ground and sieved to obtain rhizome powder. A precise amount of sample powder was weighed and subjected to constant-temperature extraction with pure water to isolate saponin components. The extract solution was centrifuged, and the supernatant was collected. Polysaccharides, pigments, and other impurities were removed using an ODS solid-phase extraction column (Solarbio, Beijing, China), thereby purifying the sample to prepare the ginsenoside test solution. The analysis employed a C18 chromatographic column (Waters, Milford, MA, USA) with a mobile phase consisting of chromatographic acetonitrile and ultrapure water, performing gradient elution at a column temperature of 30 °C, flow rate of 1.0 mL/min, detection wavelength of 203 nm, and injection volume of 10 µL. Ginsenoside quantification was performed by determining absorption peaks from a standard gradient curve. The concentrations of ginsenosides Rb1, Rg2, Rh1, Rc, Rb2, Rb3, Rd, and Rg3 were calculated based on peak areas, which were then converted into extract concentration values. The actual saponin content in tissues was determined by combining pre-treatment dilution, volumetric adjustment, and sample mass calculations. Additionally, Spearman correlation coefficients and non-parametric tests were used to analyze the correlations between saponin contents, and the Cytoscape version 3.10 software was employed to construct co-expression networks with a threshold of |r| ≥ 0.7 and *p* < 0.05.

## 3. Results

### 3.1. Comprehensive Genome Identification Analysis of PgCPP Genes in Ginseng

In the ginseng genome and transcriptome database, a hidden Markov model (HMM) with a core CXC motif (pfam03638) was used as a detection tool. After integration and comparison, 44 *PgCPP* genes were identified (Table S1). Based on their origins and annotations, these genes were named *PgCPP01*, *PgCPP02-1* to *PgCPP02-22*, *PgCPP03-1* to *PgCPP03-18*, *PgCPP04*, *PgCPP05*, and *PgCPP06*. The *PgCPP* gene length ranged from 336 to 1785 bp. The shortest gene length was 336 bp, including multiple members such as *PgCPP02-2*, *PgCPP02-10*, *PgCPP02-11*, *PgCPP02-15*, and *PgCPP02-22*, while the longest gene length was 1785 bp for *PgCPP02-12*, demonstrating diversity in molecular size within this family. The shortest encoded amino acid lengths were found in *PgCPP02-2* (111 amino acids) and *PgCPP06* (112 amino acids), while the longest was recorded in *PgCPP02-12* (594 amino acids). All *CPP* members of this gene family contain the conserved CXC domain characteristic of the CPP transcription factor family.

### 3.2. Phylogenetic Relationships and Gene Structure Analysis of PgCPP Genes

**Figure 1:**
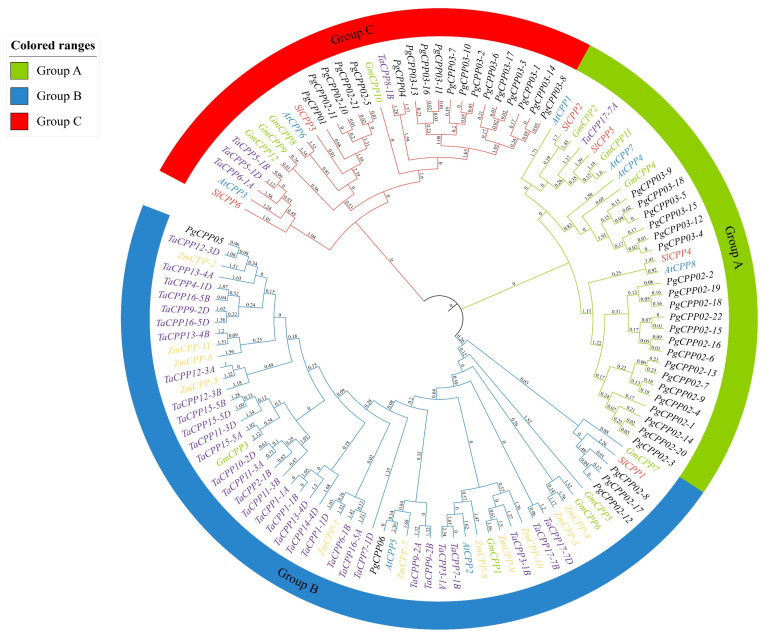
Phylogenetic relationships of the *CPP* gene family members in *P. ginseng*. This phylogenetic tree was constructed based on 74 genes from five plant species—*Arabidopsis* (At), maize (Zm), tomato (Sl), soybean (Gm), and wheat (Ta)—and the 44 *PgCPP* genes from ginseng (Pg). Different colored fonts represent different species: *CPP* genes from ginseng are shown in black, *CPP* genes from *Arabidopsis* in blue, *CPP* genes from maize in yellow, and *CPP* genes from tomato gene in red, *CPP* genes from soybean in green, and *CPP* genes from wheat in purple. This phylogenetic tree is divided into three groups, with branches represented by specific colors.

To clarify the phylogenetic and evolutionary relationships of the *CPP* gene family and systematically classify them, we screened the 44 *PgCPP* genes and 74 *CPP* genes from five other plant species. According to the phylogenetic analysis (Figure 1), the *CPP* gene family members can be divided into three major branches (Groups A, B, and C) based on their structural characteristics. Members of the *PgCPP* family are distributed across three subfamilies, exhibiting diverse evolutionary patterns. Group A includes *PgCPP02-2* and *PgCPP03-15*, which exhibit close clustering with tomato *CPP* genes and demonstrate narrow genetic distances, reflecting high inter-species sequence conservation. *PgCPP03-9* belongs to Group B, forming a clade together with soybean *GmCPP4* and *AtCPP*, indicating close phylogenetic relationships. The *PgCPP*s form the majority of Group C, including *PgCPP01*, *PgCPP02*, and the remainder of *PgCPP03*; this branch also encompasses *CPP* genes from monocots (wheat and maize) and dicots (soybean and tomato). These findings suggest varying degrees of evolutionary differentiation among members of different *PgCPP* subfamilies, with the gene family emerging prior to the divergence between monocots and dicots while maintaining relatively conserved homology throughout species evolution. Phylogenetic analyses reveal significant evolutionary relationships among *CPP* genes, which are closely associated with gene duplication and the functional diversity of their encoded proteins. 

**Figure 2:**
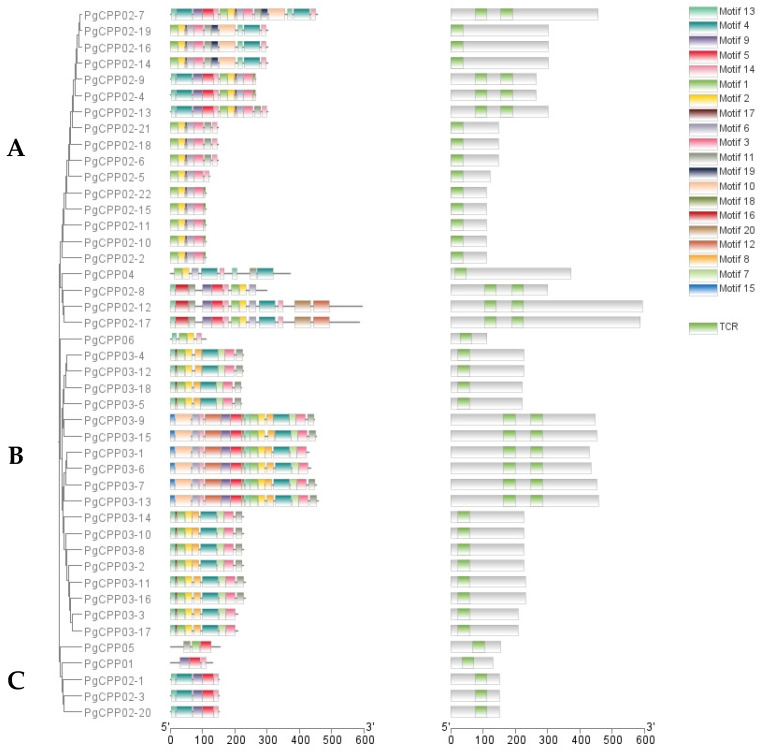
Phylogenetic tree, conserved motif, and conserved domain analysis of *PgCPP* genes. The *PgCPP* genes can be divided into three groups: A, B, and C. Boxes with different colors represent different conserved motifs or conserved domains.

To explore the genetic structural characteristics of the CPPs in ginseng, we systematically predicted and analyzed their motif elements and conserved domains. The structural analysis of PgCPPs is shown in Figure 2, in which it can be seen that the CPP family in ginseng is divided into three subfamilies. The PgCPP motifs in groups A and B are relatively complex and significantly different from those in group C. Among the 44 PgCPPs, 15 conserved motifs (denoted motifs 1–15) were identified. The TCR motif is the most conserved among them, which is crucial for determining their biological function and is present in all PgCPPs. The significant differences in the number and composition of these conserved domains among different branches may be related to the different functions of genes from each branch, providing important clues for deeper understanding of the functional differentiation among ginseng CPP family members.

### 3.3. Chromosomal Localization and Collinearity Analysis of PgCPP Genes in Ginseng

**Figure 3:**
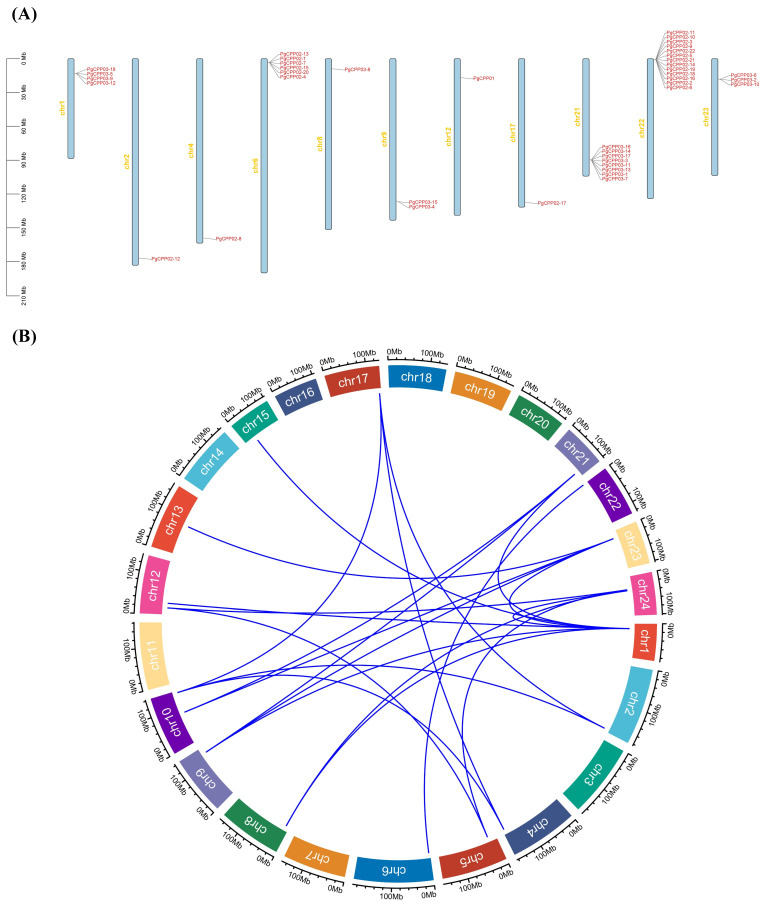
*PgCPP* gene localization and collinearity analyses in the ginseng genome. (**A**) Chromosomes that do not show gene localization are not shown, while chromosomes that show gene localization are shown in blue. (**B**) Each arc points to a paraphyletic pair resulting from gene duplication. The colored boxes represent the chromosomes of ginseng, and the scale outside each chromosome marks its length.

Following chromosome localization of the *PgCPP* gene family members, we identified 44 *PgCPP* genes, named *PgCPP01* to *PgCPP06* (Figure 3A), which are unevenly distributed on 11 chromosomes (chromosomes not genetically localized are not displayed). Furthermore, three of these members (*PgCPP04*, *PgCPP05*, and *PgCPP06*) have not yet been localized on ginseng’s 24 chromosomes. Chromosome 22 contained the most *PgCPP* genes (13 genes), chromosome 21 contained 8 genes, chromosome 6 contained 6 genes, chromosome 1 contained 4 genes, chromosome 23 contained 3 genes, chromosome 9 contained 2 genes, and 1 member was found for each of chromosomes 2, 4, 8, 12, and 17. Further isolinearity analysis revealed that 22 pairs of genes in the *PgCPP* gene family exhibited collinearity (Figure 3B); these collinear gene pairs have highly similar sequences and may be functionally conserved.

### 3.4. PgCPP Gene Family Members Expression Pattern Analysis in Ginseng

**Figure 4:**
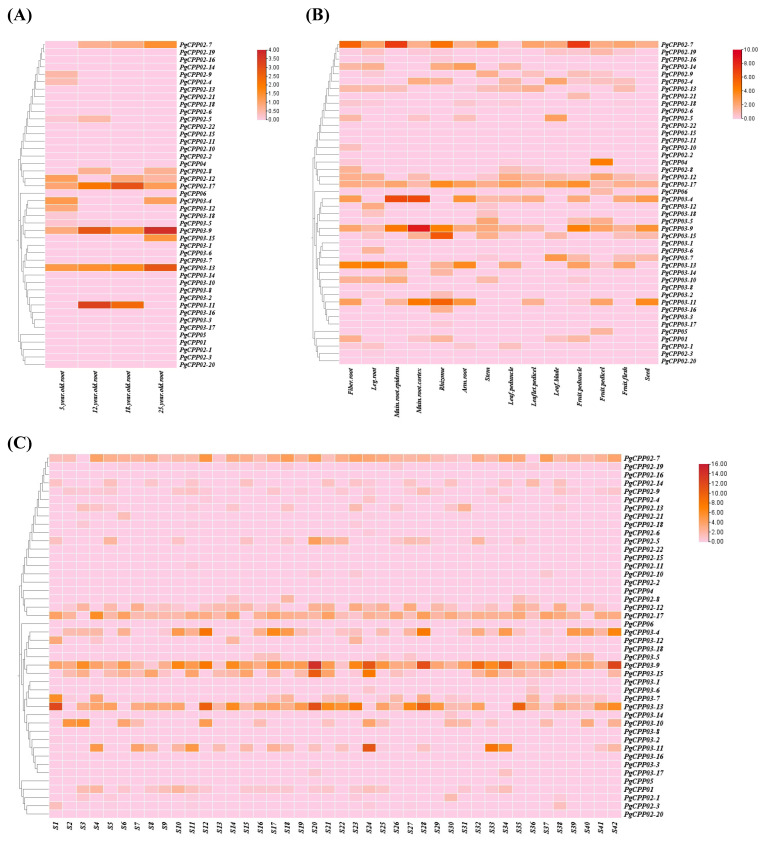
Spatiotemporal expression patterns of *PgCPP* genes in ginseng, presented as heatmaps. (**A**) *PgCPP* gene expression in ginseng roots at four different growth ages (5, 12, 18, and 25 years). (**B**) *PgCPP* gene expression in 14 different tissues of 4-year-old ginseng. (**C**) *PgCPP* gene expression in roots of 42 cultivars (4-year-old ginseng).

To investigate the expression characteristics of *PgCPP* genes in plants at different ages, tissues, and landrace ginseng cultivars, we constructed *PgCPP* gene expression heatmaps (Figure 4, Table S2). Specifically, we analyzed plants at four different ages (Figure 4A), 14 different tissues of four-year-old ginseng (Figure 4B), and 42 landrace cultivars (Figure 4C). The *PgCPP03-9* transcript showed the highest expression levels in ginseng roots at different ages, all 14 tissues of 4-year-old ginseng, and all 42 cultivars. It was upregulated with age in roots of different ages, widely expressed across multiple tissues, and persistently high in all samples, making it highly likely to participate in growth and development, stress response, and age-related secondary metabolic regulation in ginseng. *PgCPP* gene family members may play key roles in root development and overall growth. *PgCPP03-13* was upregulated in roots with age and widely expressed in multiple samples. The results revealed significant differences between the *PgCPP* genes among different tissues, growth stages, and cultivars, which are likely closely related to the physiological needs, metabolic activities, and genetic background of ginseng at different growth stages.

### 3.5. PgCPP Gene Co-Expression Network Analysis

**Figure 5:**
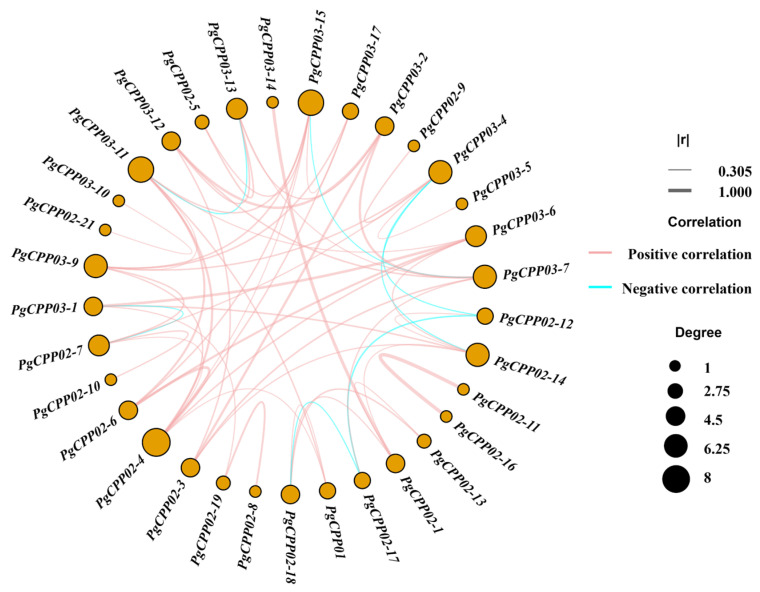
Correlation network diagram of *PgCPP* gene family members at the significance level of *p* < 0.05.

To explore the correlations between the expression of *PgCPP* genes, this study analyzed the 44 *PgCPP* genes (including *PgCPP04*, *PgCPP05*, and *PgCPP06*) through co-expression network analysis (Figure 5). At a saliency level of *p* ≤ 0.05, a co-expression network containing 32 nodes and 52 edges was constructed. *PgCPP03-15* was the highest core hub gene in the network, with strong and significant correlations among multiple members. Genes such as *PgCPP02-4*, *PgCPP03-9*, and *PgCPP03-11* also had high connectivity, forming a secondary regulatory core in the network. These pivotal genes play key regulatory roles in the network and may be key drivers of functional execution in the *PgCPP* family. 

### 3.6. Cis-Acting Element Promoter Analysis of PgCPP Members

**Figure 6:**
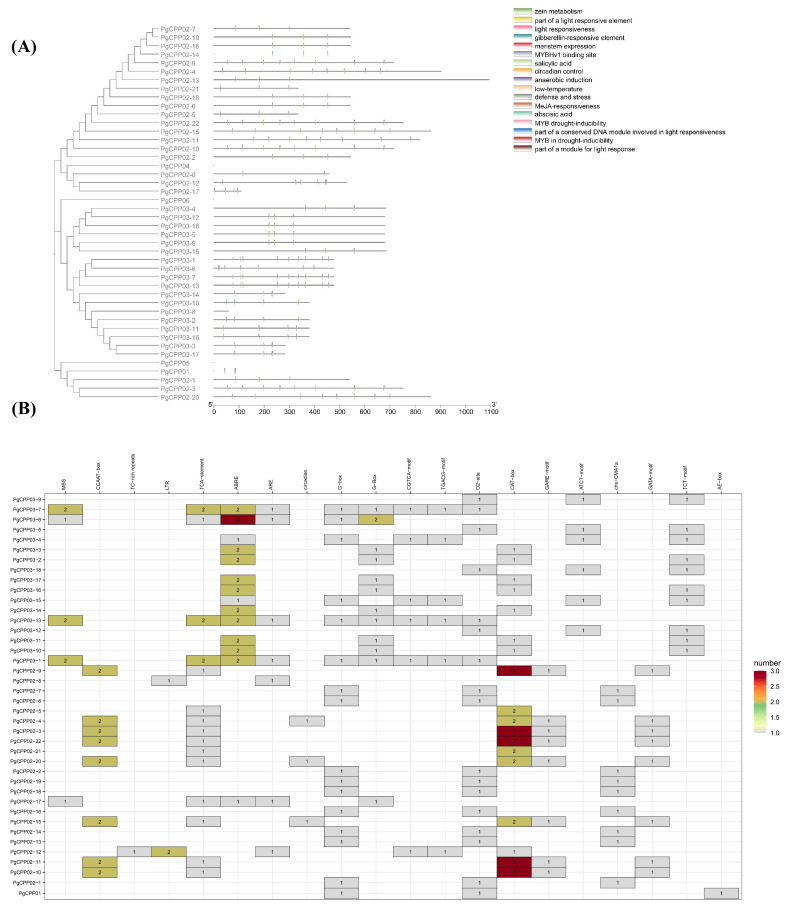
Cis-acting element promoter analysis of *PgCPP* gene family members. (**A**) Cis-acting element composition analysis in the *PgCPP* gene family members. Different colored circles represent cis-acting elements with different functions. (**B**) Heatmap of the cis-acting elements in the *PgCPP* gene family members.

Cis-acting elements play a key role in the molecular mechanisms regulating the functions of *PgCPP* genes. This study conducted a systematic and comprehensive analysis of the promoter region of the *PgCPP* genes, revealing up to 17 types of cis-acting elements (Figure 6). The promoter regions of all *PgCPP* genes contained various types of cis-acting elements, divided into four functional categories: light response, hormone signal regulation, stress response, and tissue development regulation (Figure 6A). Comparing the functions of the cis-acting elements present in *CPP* genes across various plants, it was found that the promoters of *SlCPP1*, *SlCPP3*, and *SlCPP4* contain multiple light-responsive cis-acting elements, which exhibit a significant upregulation under strong light stress and are involved in the oxidative photodamage scavenging pathway. Furthermore, light signals and drought stress have been reported to exert cross-regulatory effects on the expression of *SlCPP* genes [23]. The *TaCPP* gene family in wheat responds to various signaling molecules, including IAA, ETH, and ABA. Overexpression of *TaCPP5-1D* altered endogenous ABA levels and regulated root and shoot growth via hormonal pathways [6]. In this study, we identified various cis-acting elements with different functions, including protein binding sites, zea-isolate protein metabolism regulatory elements, and trauma response elements (Figure 6B). These cis-acting elements exhibited a high degree of diversity in both type and quantity, indicating that the *PgCPP* gene family may possess broad functional diversity during growth, development, and environmental adaptation. The promoter regions of the majority of *PgCPP* genes encompass cis-acting elements associated with hormone responses. These elements are categorized into five groups, corresponding to the signal responses to methyl jasmonate (MeJA), abscisic acid (ABA), auxin, gibberellin (GA), and salicylic acid (SA). Notably, MeJA response elements exhibited the highest abundance among all hormone response elements, indicating that the *PgCPP* gene family members are significantly involved in hormone signaling pathways and play crucial regulatory roles. The *PgCPP* genes *PgCPP03-7*, *PgCPP02-3*, *PgCPP03-13*, and *PgCPP03-9* contained the most MeJA regulatory elements within their promoter regions. As such, these genes exhibit strong MeJA signal-sensing capabilities and can be activated under suitable external signal induction, thereby contributing to the stress response in ginseng. Furthermore, the promoter regions of these four genes contain both defense elements and multiple stress response elements, suggesting that their functions may involve the cross-regulation of multiple signaling pathways, thereby enhancing the complexity and diversity of their stress resistance regulatory roles in ginseng.

### 3.7. Screening of PgCPP Candidate Genes Related to Ginsenoside Biosynthesis

**Figure 7:**
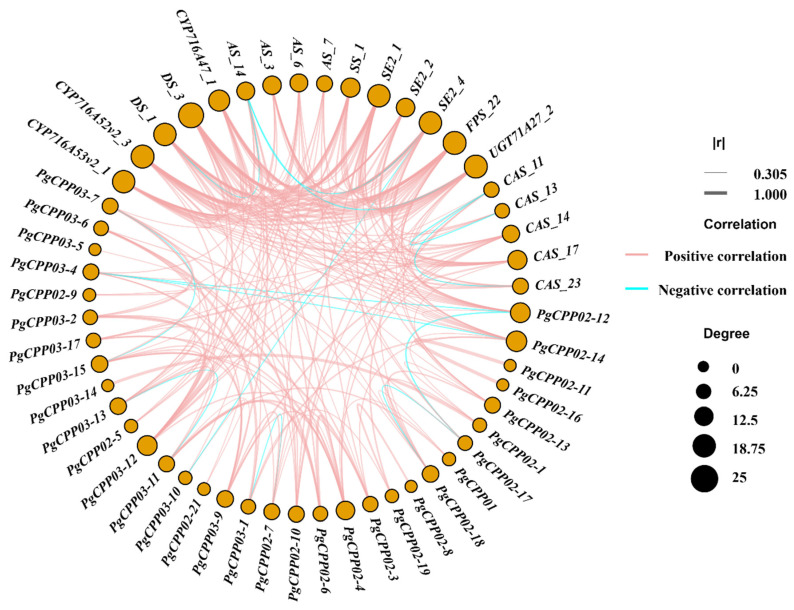
Correlation interaction network diagram between *PgCPP* genes and key enzyme genes in ginsenoside biosynthesis. The *p* < 0.05 and the correlation threshold is 0.305.

To explore the potential regulatory relationships between the *PgCPP* gene family and ginsenoside biosynthesis pathways, we constructed a co-expression correlation network (*p* < 0.05) between *PgCPP* genes and key enzyme genes in ginsenoside synthesis (Figure 7). The results revealed that most *PgCPP* genes were associated with key enzyme genes of ginsenoside synthesis (e.g., *SS_1*, *SE2_1*, *CAS_17*, and *CYP716A4_1*), with only a small number of gene pairs showing significant negative correlations, indicating that members of the *PgCPP* gene family mainly participate in the biosynthesis of ginsenosides through co-regulatory patterns. Based on linkage analysis, *PgCPP03-9* was identified as the core gene in this co-expression correlation network, showing broad and significant correlations with several key enzyme genes in ginsenoside synthesis; therefore, this gene may play a crucial role in the transcriptional regulation of ginsenoside synthesis. Furthermore, *PgCPP02-7*, *PgCPP02-4*, and *PgCPP03-1* exhibited high connectivity, forming secondary core junctions in the regulatory network. Comparative analyses at different subfamily levels showed that members of the *PgCPP03* subfamily had significantly higher overall correlations with key enzyme genes of ginsenoside synthesis, when compared to those in the *PgCPP02* subfamily, further confirming the dominant role of this subfamily in regulating ginsenoside synthesis. These results provide important genetic resources and a theoretical foundation for further analysis of the molecular mechanisms by which *PgCPP* gene family members regulate ginsenoside biosynthesis.

### 3.8. PgCPP Gene Expression Analysis Under MeJA Treatment

**Figure 8:**
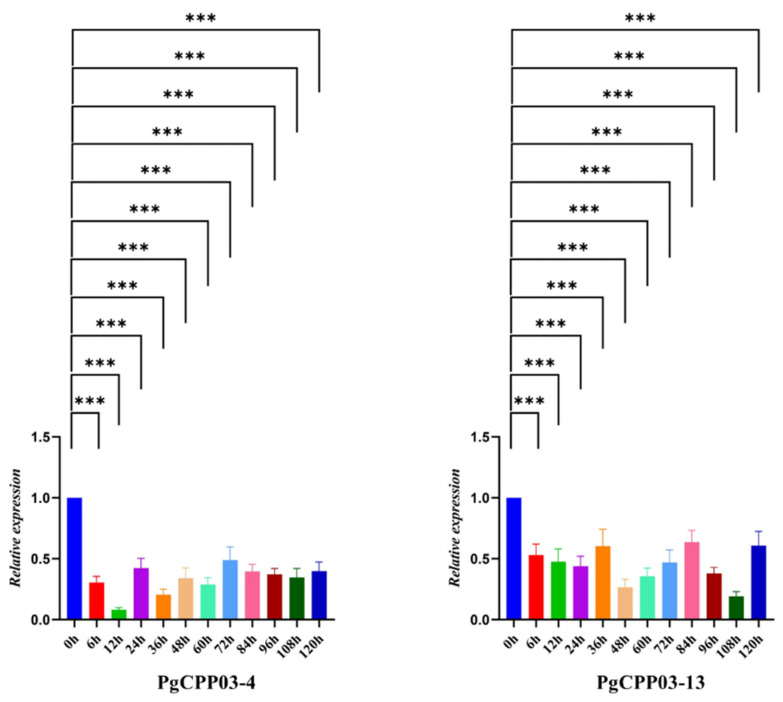
Expression analysis of *PgCPP* candidate genes after MeJA treatment. Significance analysis plot of the expression levels of the *PgCPP03-4* and *PgCPP03-13* genes (mean ± standard deviation). The relative expression levels of the two *PgCPP* genes were calculated with respect to the gene expression level at 0 h (set to 1). The value is the average of the three experimental replicates. *** indicates a significant difference at *p* ≤ 0.001.

**Figure 9:**
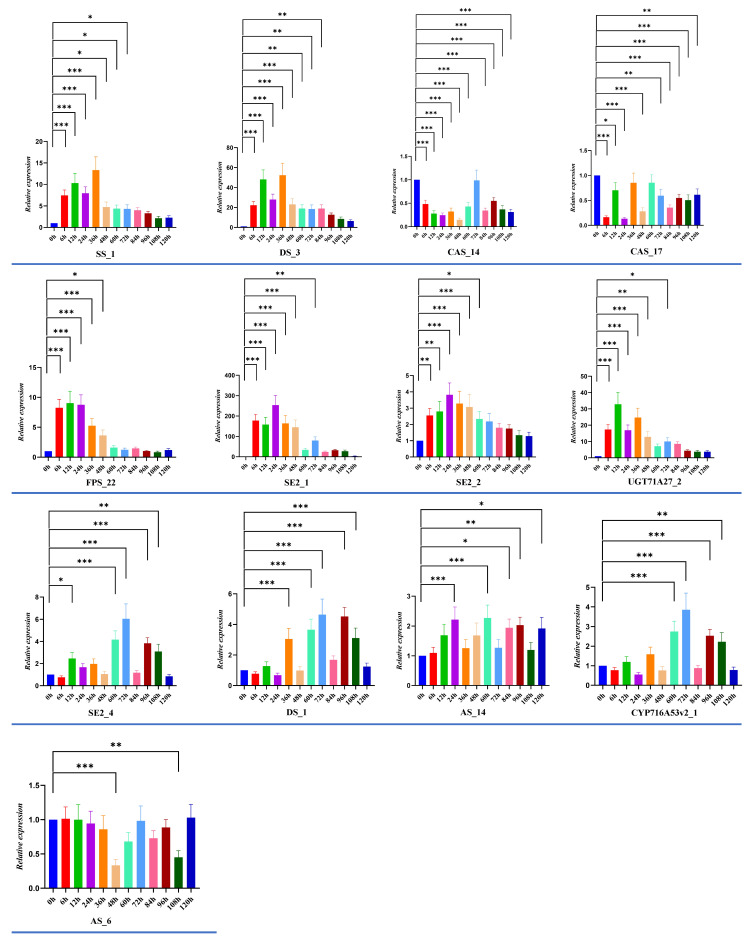
Expression analysis of key enzyme genes in ginsenoside synthesis after MeJA treatment. The expression levels of key enzyme genes (*CAS_17*, *SS_1*, *DS_3*, *SE2_1*, *UGT71A27_2*, *SE2_2*, *AS_14*, *CYP716A53v2_1*, *DS_1*, *FPS_22*, *SE2_4*, *AS_6*, and *CAS_14*) are shown as the mean ± standard deviation. The relative expression levels of these 13 genes were calculated with respect to the gene expression level at 0 h (set to 1). The value is the average of the three replicates of the experiment. * indicates a significant difference at *p* ≤ 0.05, ** indicates a significant difference at *p* ≤ 0.01, *** indicates a significant difference at *p* ≤ 0.001.

To clarify the regulatory role of MeJA on the *PgCPP* genes and ginsenoside synthesis pathways, this study used the ginseng adventitious root as a plant material, focusing on two *PgCPP* genes (*PgCPP03-4* and *PgCPP03-13*) and key enzyme-encoding genes (*CAS_17*, *SS_1*, *DS_3*, *SE2_1*, *UGT71A27_2*, *SE2_2*, *AS_14*, *CYP716A53v2_1*, *DS_1*, *FPS_22*, *SE2_4*, *AS_6*, and *CAS_14*) (Figure 8 and Figure 9). Although the co-expression network analysis also identified *PgCPP03-9* and *PgCPP03-15* as core hub genes, the promoter cis-element analysis revealed that *PgCPP03-13* contains both MeJA and defense/stress response elements, while the correlation analysis demonstrated that the expression levels of *PgCPP03-4* and *PgCPP03-13* exhibited the most significant negative correlations with the concentrations of the six pro-ginsenosides (*p* < 0.05). The results showed that all 15 genes responded to MeJA induction, to varying degrees. Specific analyses showed that *PgCPP03-4* and *PgCPP03-13* exhibited significant responses to MeJA induction, characterized by downregulated expression levels. The relative expression level of *PgCPP03-4* continuously decreased over 12 h, reaching its lowest value at 12 h after treatment. However, starting from the 24 h period, although there were slight fluctuations, the overall expression level gradually rebounded and remained below that of the control group. In contrast, *PgCPP03-13* exhibited a fluctuating expression pattern, being significantly downregulated at 36, 84, and 120 h, and consistently lower than in the CK group, showing a downregulated expression pattern. Regarding key enzyme genes, throughout the entire 120 h experiment, the relative expression levels of *CAS_17* and *CAS_14* in the treatment group were lower than in the CK group. *PgCPP03-4* and *PgCPP03-13* were significantly downregulated in a manner similar to the key enzymes *CAS_17* and *CAS_14*. These results indicate that, under MeJA induction, *PgCPP* genes involved in ginsenoside biosynthesis may be downregulated, with their expression patterns showing significant differences from those of the key enzyme genes.

### 3.9. Correlation Between PgCPP Gene Expression and Protopanaxadiol-Type Ginsenoside Contents

**Figure 10:**
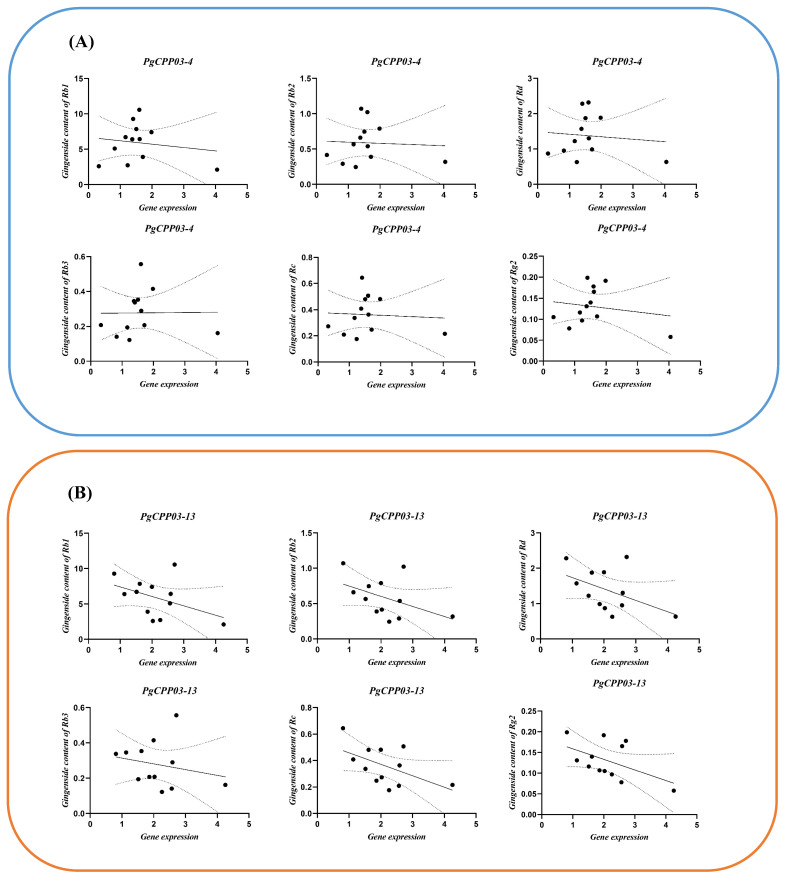
Correlation analysis between *PgCPP03-4* and *PgCPP03-13* gene expression levels and Rb1, Rb2, Rc, Rd, Rb3, and Rg2 ginsenoside contents. (**A**) Correlations between the expression level of the *PgCPP03-4* gene and the contents of six ginsenosides. (**B**) Correlations between the expression level of the *PgCPP03-13* gene and the contents of six ginsenosides. The dotted line represents the 95% confidence interval (CI), the solid line represents the linear regression fitting curve, and each black point represents a single sample observation.

This study systematically compared the expression levels of *PgCPP03-4* and *PgCPP03-13* and the alterations in protopanaxadiol-type ginsenoside contents after MeJA treatment. These findings indicated a significant negative correlation between the expression levels of these *PgCPP* genes and the content of protopanaxadiol-type ginsenosides (Figure 10). Following treatment, the *PgCPP03-13* gene and protopanaxadiol-type ginsenoside content exhibited marked declines, whereas the decrease in *PgCPP03-4* gene expression was less pronounced. Further analysis suggested that this negative correlation may be closely associated with MeJA-related activation of the stress response signaling pathway in ginseng. Previous transcriptomic studies have demonstrated that MeJA treatment of ginseng adventitious roots activates a well-defined jasmonic acid (JA) stress signaling pathway, which transmits stress signals hierarchically through the COI1–JAZ–MYC core module to broadly regulate the expression of genes involved in secondary metabolism [24]. Similarly, it can be inferred that induction with MeJA likely triggers a stress-related signaling cascade that regulates the expression of *PgCPP* genes. This interaction may influence the expression of key genes in ginsenoside biosynthetic pathways, ultimately resulting in a reduction in protopanaxadiol-type ginsenoside content. This discovery elucidates a potential regulatory mechanism linking *PgCPP* gene expression with MeJA-induced ginsenoside biosynthesis, offering new insights into the regulation of secondary metabolism in ginseng.

## 4. Discussion

Ginseng (*Panax ginseng* C. A. Meyer) is an important medicinal plant in the Araliaceae family, which is renowned worldwide for its outstanding pharmacological activity. Based on the ginseng genome and transcriptome database, this study identified 44 *PgCPP* genes in ginseng. Compared with maize [2] and tomato [25], *PgCPP* genes are more abundant, possibly because ginseng is tetraploid and has undergone gene replication events during its evolution. Similar phenomena have also been observed in alfalfa (31 *MsCPP* genes) [4] and tea (10 *CsCPP* genes) [26], indicating significant differences in the number of *CPP* gene family members among plants from different regions, which are closely related to genomic ploidy and evolutionary history. Chromosomal localization demonstrated that *PgCPP* genes are unevenly distributed on ginseng’s chromosomes, with chromosome 22 containing 13 members; furthermore, some of the *PgCPP* genes have not yet been localized. The distribution of the *MdCPP* gene family in apple presents similar characteristics [27]. In the collinearity analysis, we identified 22 pairs of collinear genes, suggesting gene recombination or fragment duplication during family expansion. Phylogenetic analysis classified the 44 *PgCPP* genes into three branches—A, B, and C—with branch A containing the largest number of members, resulting in significant expansion of this branch. Similarly, the *CPP* families of tea and alfalfa have been divided into multiple subgroups, where the differences between species correspond to their functional differentiation. From the gene structure, it can be seen that the A subfamily has a more complex motif composition, potentially providing the molecular basis for this functional diversity. Promoter analysis revealed that the *PgCPP* gene family members contain up to 17 types of cis-acting elements in the promoter region involved in responses to light, various hormones (e.g., MeJA, ABA, auxin), and abiotic stress. Among them, MeJA response elements had the highest abundance, indicating that *PgCPP* genes may be deeply involved in the MeJA signaling pathway. Among them, the promoters of genes *PgCPP03-7*, *PgCPP02-3*, *PgCPP03-13*, and *PgCPP03-9* simultaneously contained MeJA and defense/stress elements, suggesting their multifunctional roles in stress resistance and secondary metabolic regulation. Similar results have been reported for apples, alfalfa, and tea trees. Previous studies have shown that MeJA regulates secondary metabolism through transcription factor families such as MYB [12] and WRKY [13]. This study identified *PgCPP* genes rich in MeJA response elements, suggesting that the *CPP* gene family members may serve as new genes involved in the regulatory network of ginsenosides. Notably, recent advancements in single-cell transcriptomics and spatial transcriptomics have provided cell-type-specific insights into the regulatory mechanisms of plant stress responses [28]. Leveraging these novel techniques, it may be possible to further precisely elucidate the regulatory network of the *PgCPP* genes across different ginseng cell types, thereby identifying more precise molecular targets and offering new approaches for subsequent functional validation.

Research has shown that MeJA, as a classic plant hormone signaling molecule, plays a key role in the transcriptional regulation of secondary metabolites in medicinal plants [29], and has been shown to significantly promote the accumulation of various active medicinal compounds, including notoginseng saponins, tanshinone, and paclitaxel [30]. The Cys-rich Polycomb-like Protein (CPP) family is a class of plant-specific transcription factors containing conserved CXC domains, which have been confirmed to be involved in processes such as plant growth and development, stress response, and hormone signal transduction. However, the systematic identification of ginseng *CPP* gene family members and their functions in the MeJA signaling pathway remain understudied. In this study, MeJA was used to treat the adventitious roots of ginseng, following which the expression changes in *PgCPP03-4*, and *PgCPP03-13*, as well as key enzyme genes related to ginsenoside biosynthesis were detected. The results demonstrate that neither of these *PgCPP* genes were upregulated after MeJA treatment; instead, they exhibited sustained downregulation or fluctuating suppression (with expression levels remaining below the control level). Key enzyme genes involved in ginsenoside biosynthesis (e.g., *CAS_17* and *CAS_14*) also displayed similar inhibitory trends, preliminarily suggesting that *PgCPP* genes may be considered as candidate genes associated with ginsenoside synthesis. This hypothesis is supported by the synchronous downregulation of *PgCPP* genes and key enzyme genes following MeJA treatment, indicating that silencing these *PgCPP* genes may alleviate certain inhibitory effects on saponin synthesis. Furthermore, the negative correlations between gene expression levels and saponin contents align precisely with the characteristics of candidate genes exhibiting negative correlations. In medicinal plants, such as in the root of *Polygonum multiflorum*, the number of downregulated genes after MeJA treatment far exceeds the number of upregulated genes, which demonstrates the complexity of MeJA regulation to some extent [31]. The correlation analysis also revealed that the expression levels of the *PgCPP03-4* and *PgCPP03-13* genes were negatively correlated with the contents of six protopanaxadiol-type ginsenosides (Rb1, Rc, Rb2, Rd, Rg2, and Rb3); that is, increased *PgCPP* gene expression significantly decreased ginsenoside levels. Based on the available data, we hypothesize that this negative correlation may be associated with the redistribution of metabolic flows in the stress response signaling pathway induced by MeJA.

Given the differences in *PgCPP* gene responses to MeJA, different genetic transformation strategies can be used to further investigate their functions. For genes with downregulated expression (e.g., *PgCPP03-4*), the CRISPR/Cas9 gene knockout technique can be employed to elucidate their mechanisms of action [32]. Genes demonstrating core regulatory potential in network analysis (e.g., *PgCPP03-9* and *PgCPP03-15*, whose core regulatory functions were confirmed through network analysis) can further be validated via overexpression experiments to determine their involvement in saponin synthesis. The responses of *MsCPP* genes to stress conditions in alfalfa have been demonstrated using qRT-PCR technology [4], indicating the high verifiability of *CPP* gene family functions. This study systematically identified ginseng *CPP* gene family members for the first time, comprehensively revealing their genetic structure, evolutionary relationships, and distribution patterns of promoter cis-regulatory elements, while identifying key candidate genes involved in the MeJA response. These findings provide a theoretical foundation and genetic resources for molecular breeding of ginseng and may serve as a basis for advancing research on transcription factor families in other medicinal plants. Future investigations into the functions and regulatory mechanisms of *PgCPP* genes are expected to fill gaps in our understanding of the role of the *CPP* gene family in ginsenoside biosynthesis regulation.

## 5. Conclusions

In this study, 44 *PgCPP* gene family members in ginseng were identified and comprehensively analyzed, which was followed by joint expression analysis of the *PgCPP* and key enzyme genes involved in protopanaxadiol-type ginsenoside biosynthesis in response to MeJA treatment. Promoter analysis showed that the *PgCPP* genes are rich in MeJA response elements; however, after MeJA treatment, the expression of *PgCPP03-4* and *PgCPP03-13* were inhibited. Furthermore, their expression levels were significantly negatively correlated with the contents of six protopanaxadiol-type ginsenosides, suggesting that members of this family may act as negative regulators of ginsenoside synthesis in the MeJA signaling pathway. This highlights the potential of molecular breeding or genetic engineering strategies targeting specific *PgCPP* genes to regulate ginsenoside yields in medicinal plants. In the future, we will conduct in-depth research on the differential expression of *PgCPP03-4* and *PgCPP03-13* under MeJA treatment, in order to elucidate the molecular mechanisms underlying ginsenoside biosynthesis. Specifically, functional validation of *PgCPP03-4* and *PgCPP03-13* using overexpression or gene-editing technologies could be performed to confirm their definitive regulatory roles in ginsenoside biosynthesis.

## Figures and Tables

**Table 1:** Details of primers used in this study for fluorescence quantitative PCR.

Number	Primer Name	Primer Sequences	Product Size
1	Action 1-F	TGGCATCACTTTCTACAACG	150 bp
	Action 1-R	TTTGTGTCATCTTCTCCCTGTT	
2	PgCPP03-4-F	GGGGGCGATCCAATGTTACA	133 bp
	PgCPP03-4-R	ATATGCAAACTGCCCGGGAA	
3	PgCPP03-13-F	ACACAGTAGACACCAGCAGC	98 bp
	PgCPP03-13-R	GCTGGAGATAGTGAGACGGC	
4	SS_1-F	TGGGGGCAATTCTGAAGCAT	160 bp
	SS_1-R	TGTTGAATGACGAGGCCGAA	
5	DS_3-F	AGTATCCACTTCCGGCCTCT	150 bp
	DS_3-R	TGAGGATGGTGGATGGGGAT	
6	CAS_14-F	GATGGACAAGGGGCAATGGA	143 bp
	CAS_14R	ATCTCTGGGGGCAGAGGATT	
7	CAS_17-F	TGTGGTGTCACTGTCGGATG	159 bp
	CAS_17-R	TCGCACAAAGGTTACGAGCT	
8	FPS_22-F	CTGGATTGCTTTGGTGCACC	166 bp
	FPS_22-R	CTTTTGCTACAGAGGCCGGA	
9	SE2_1-F	GTCCGACAGAGCAACAGTCA	113 bp
	SE2_1-R	ACAGAAGCATGCCAGCTGAT	
10	SE2_2-F	TGTCCGACAGAGCAACAGTC	114 bp
	SE2_2-R	ACAGAAGCATGCCAGCTGAT	
11	UGT71A27_2-F	AGGTGCAGAGAGTCAAAGGC	169 bp
	UGT71A27_2-R	CAAGGATCACAGCAGCCTCA	
12	SE2_4-F	TAATCAAAGCATGCGTCGCG	80 bp
	SE2_4-R	TTGGCTGGTGCGCTATACAA	
13	AS_14-F	TGCTAAAGCCCACAACGGAT	189 bp
	AS_14-R	ATGGGCCTGATTCACACTGG	
14	CYP716A53v2_1-F	CGCTGACTTTTGAGTTGGCC	155 bp
	CYP716A53v2_1-R	GGCCTTGATCCCACGGTTAA	
15	AS_6-F	TGAAGCACACACCCCAGTTT	164 bp
	AS_6-R	AGAGTGCACTTCATCGGCAA	

## Data Availability

The data used in this study are available at the Sequence Read Archive (SRA) of the National Center for Biotechnology Information (NCBI) of BioProject PRJNA302556. All ginseng samples are available upon request from the corresponding author.

## Supplementary Materials

The following supporting information can be downloaded at: https://www.mdpi.com/article/10.3390/biology15131063/s1, Table S1. Basic sequence information of *PgCPP* gene family members in ginseng; Table S2. TPM expression of the *PgCPP* genes in 42 cultivars, 14 tissues, and 4 aged roots in ginseng.
